# Flexible ureterorenoscopy and lithotripsy with pulsed thulium: YAG laser: a multicenter retrospective study

**DOI:** 10.1007/s00345-026-06467-1

**Published:** 2026-05-24

**Authors:** Carlos González González, Jia-Lun Kwok, Pietro Scilipoti, Federico Zorzi, Laurent Berthe, Nicola Nannola, Hubert Werth, Chicaud Marie, Stefano Moretto, Juan Manuel López Martínez, Steeve Doizi, Olivier Traxer, Mariela Corrales, Alba Sierra, Frédéric Panthier

**Affiliations:** 1 Endolase Lab, GRC n°20-Sorbonne Université, PIMM Lab Arts et Métiers ParisTech, 75020 Paris, France; 2 Service d’Urologie, Assistance-Publique Hôpitaux de Paris, Hôpital Tenon, Sorbonne Université, 75020 Paris, France; 3 PIMM, UMR 8006 CNRS-Arts et Métiers ParisTech, 151 bd de l’Hôpital, F-75013 Paris, France; 4 Department of Urology, Tan Tock Seng Hospital, Singapore, Singapore; 5 Lee Kong Chian School of Medicine, Nanyang Technological University, Singapore, Singapore; 6 Progressive Endourological Association for Research and Leading Solutions (PEARLS), Paris, France; 7 Section of Endourology, European Association of Urology, Arnhem, Netherlands; 8 Department of Experimental Oncology, Unit of Urology, URI, IRCCS Ospedale San Raffaele, Milan, Italy; 9 Vita-Salute San Raffaele University, Milan, Italy; 10 Service d’urologie, CHU de Limoges, 2 avenue Martin Luther King, 87000 Limoges, France; 11 Department of Urology, Hospital Clínic de Barcelona, Barcelona, Spain; 12 Service d’urologie, CHU Gabriel-Montpied, 58, rue Montalembert, 63000 Clermont-Ferrand, France; 13 Endourology Technology Section of European Association of Urology (EAU), Arnhem, The Netherlands

**Keywords:** Pulsed thulium YAG, Ureterorenoscopy, Laser, Lithotripsy, Volume, Multicenter, Stone composition

## Abstract

**Introduction:**

In vivo data on volumetric laser efficiency and clinical outcomes of the p-Tm: YAG laser from large multicenter cohorts remain limited. The present study aimed to evaluate the real-world in vivo volumetric ablation efficiency of the p-Tm: YAG laser during flexible ureterorenoscopy (FURS), with secondary assessment of clinical outcomes and safety in a multicenter cohort.

**Methods:**

We conducted a retrospective multicenter cohort study including adult patients (≥ 18 years) who underwent p-Tm: YAG–based FURS for ureteral and/or renal calculi at three tertiary referral centers between 2023 and 2025. Preoperative stone volume was quantified using manual three-dimensional CT segmentation. Laser-on time (LOT), total delivered energy, and laser settings were prospectively recorded. Volumetric ablation speed (mm³/s) and energy consumption (J/mm³) were calculated using standardized definitions based on segmented pre- and postoperative stone volumes in patients with postoperative non-contrast CT (NCCT). Stone-free rate (SFR) was assessed 1–3 months postoperatively using NCCT and classified as Grade A (no residual fragments), Grade B (residual fragments ≤ 2 mm), and Grade C (residual fragments ≤ 4 mm). In patients without postoperative NCCT, SFR was assessed by intraoperative endoscopic evaluation and analyzed separately.

**Results:**

Of 222 screened patients, 167 met the inclusion criteria. Median preoperative stone volume was 987 mm³ (IQR 329–3,124), and 47% of patients presented with multiple/complex stones. Median LOT was 21.4 min, and median total delivered energy was 12 kJ. In the 108 patients with paired volumetric data and postoperative NCCT, median ablation speed was 0.55 mm³/s (IQR 0.30–0.95), and median energy consumption was 13 J/mm³ (IQR 8–23). In this NCCT subgroup, SFR were 47%, 59%, and 66% for Grade A, B, and C, respectively. Among the 59 patients assessed endoscopically only, complete stone clearance was visually assessed in 20 patients (34%). Overall, 45 patients (27%) required additional stone-related treatment. Complications were uncommon, with 4.8% Clavien–Dindo grade I–II events and 0.6% grade III events; no grade IV–V complications were observed.

**Conclusion:**

In this multicenter real-world cohort, p-Tm: YAG laser lithotripsy showed measurable volumetric efficiency and was associated with acceptable stone clearance and a favorable safety profile during FURS. These findings support the clinical feasibility of this technology in routine endourological practice; however, prospective comparative studies are warranted to further define its relative performance compared with established laser platforms.

**Supplementary Information:**

The online version contains supplementary material available at 10.1007/s00345-026-06467-1.

## Introduction

Urinary stone disease affects 5–12% of the global population, with a steadily increasing incidence attributed to lifestyle, metabolic, and dietary factors [1]. Flexible ureterorenoscopy (FURS) with endoscopic laser lithotripsy has become a cornerstone treatment for ureteral and renal calculi, offering high stone-free rates (SFR) with low morbidity [2]. According to current international guidelines, Holmium: yttrium–aluminum–garnet (Ho: YAG) and thulium fiber laser (TFL) represent the standard energy sources for endoscopic laser lithotripsy due to their effectiveness across a wide range of stone compositions and anatomical locations [1].

The Ho: YAG laser has historically been considered the gold standard for endoscopic laser lithotripsy; however, its high peak power and short pulse duration may contribute to increased retropulsion and reduced dusting efficiency [3]. In contrast, the TFL has demonstrated improved dusting capability and ablation efficiency due to its lower peak power, higher pulse frequencies, and more favorable pulse shape characteristics [4].

The pulsed thulium: yttrium–aluminium–garnet (p-Tm: YAG) laser is a newer solid-state platform introduced to combine advantageous features of both Ho: YAG and TFL technologies [5]. Operating at a wavelength of approximately 2013 nm and capable of delivering peak powers up to 3.7 kW, the p-Tm: YAG allows a wide range of pulse durations, frequencies, and energy settings, enabling both dusting and fragmentation strategies [6]. Preclinical studies suggest that p-Tm: YAG achieves ablation efficiency comparable to TFL while producing less retropulsion than Ho: YAG [7–9]. Early clinical experiences further support its potential, reporting SFR of reaching up to 95% in FURS with high safety profile [10].

However, real-world multicenter evidence remains limited, particularly regarding objective volumetric efficiency parameters such as ablation speed and energy consumption measured in vivo across heterogeneous stone burdens and anatomical scenarios [11]. Therefore, the present multicenter retrospective study was designed to primarily evaluate the real-world in vivo volumetric laser efficiency of the p-Tm: YAG laser during FURS. Secondary objectives included the assessment of SFR outcomes and perioperative safety in order to contextualize laser performance in routine clinical practice.

## Methods

### Study design and participants

This retrospective multicenter cohort study was conducted across three tertiary referral endourology centers: Hôpital Tenon (Paris, France), Tan Tock Seng Hospital (Singapore), and Hospital Clínic de Barcelona (Barcelona, Spain). The study included consecutive patients treated with pulsed thulium: YAG (p-Tm: YAG, Thulio^®^, Dornier MedTech, Wessling, Germany) laser lithotripsy and was conducted in accordance with national and institutional research regulations.

Data processing was declared to the Commission Nationale de l’Informatique et des Libertés (CNIL) under the MR-004 framework (approvals 2216615 v0 and 2230198 v0). Ethical approval was granted by the Comité d’Éthique et de la Recherche de l’Association Française d’Urologie (CERU approvals CERU_2020/003 and CERU_2023018B). Institutional review board approval was obtained at each participating site, and informed consent was waived or obtained in accordance with local ethics policies.

Eligible participants were adult patients (≥ 18 years) who underwent FURS with p-Tm: YAG laser lithotripsy for renal and/or ureteral calculi between January 2023 and September 2025. Patients were identified through prospectively maintained institutional surgical databases at each center. All participating institutions applied consistent preoperative evaluation protocols and standardized inclusion and exclusion criteria throughout the enrollment period to ensure cohort uniformity.

**Fig. 1:**
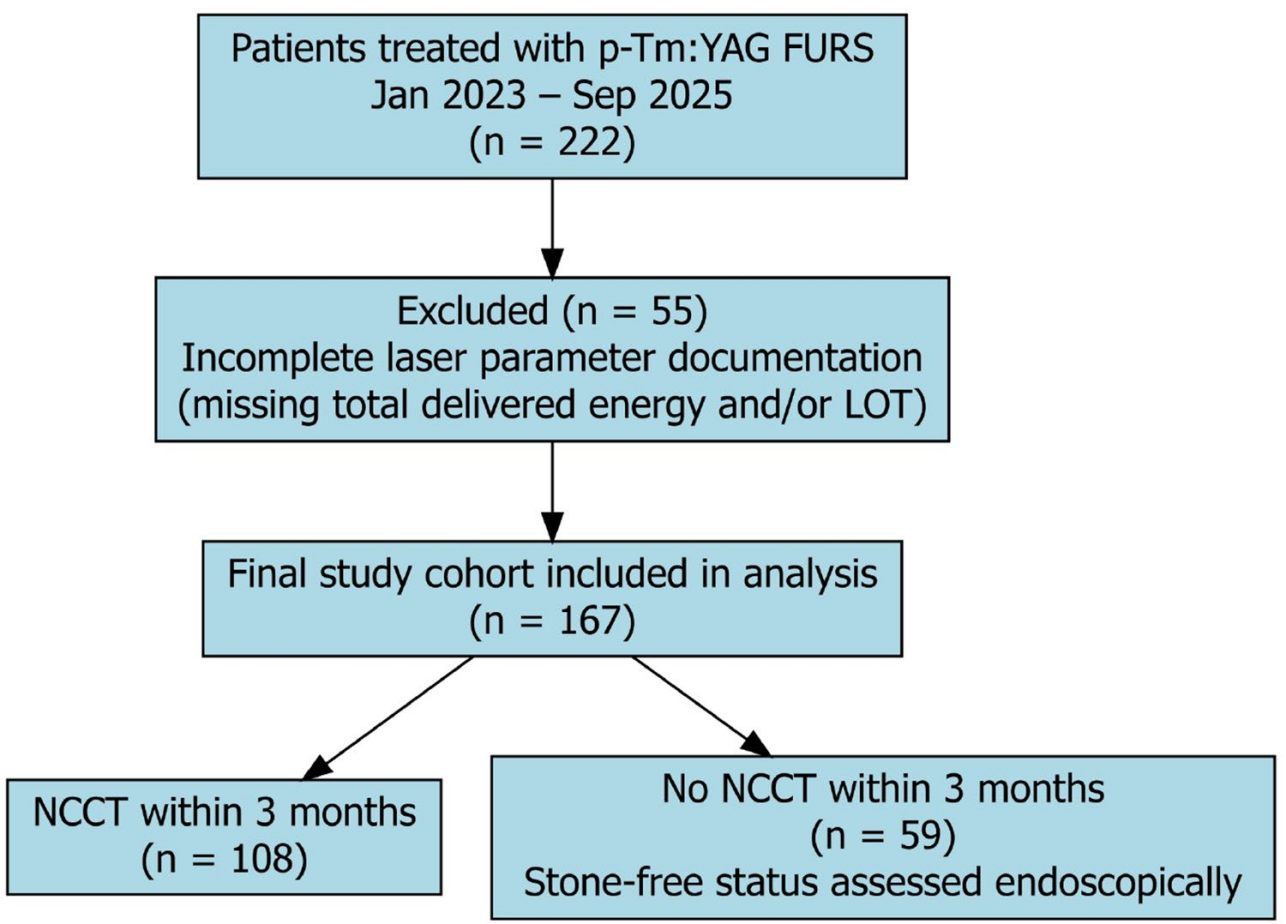
Flow diagram of patient selection. Selection of patients included in the multicenter retrospective cohort study

A total of 222 consecutive eligible patients were identified. Patients were excluded only when complete intraoperative laser data (e.g., total delivered energy and laser-on time), were unavailable, as these variables were required for volumetric efficiency analyses. Fifty-five patients were excluded due to incomplete laser parameter documentation, resulting in a final study cohort of 167 patients included in the analysis. The patient selection process is summarized in (Fig. 1).

### Surgical procedure

Flexible ureterorenoscopy was performed under general anesthesia using ureteroscopes with an outer diameter ranging from 6.3 to 9.5 Fr, including both reusable and single-use platforms.

When required, a ureteral access sheath (9.5–12 Fr) was inserted to facilitate repeated access and optimize irrigation. In selected cases, a flexible suction access sheath (FANS) was used to provide active outflow and assist with intrarenal pressure control.

Laser lithotripsy was performed using 200–272 μm laser fibers in dusting or fragmentation settings according to standard clinical practice. Maximum laser power was limited to 20 W for intrarenal lithotripsy and 10 W for ureteral stones.

Irrigation was delivered using 0.9% saline solution at room temperature. In standard procedures, irrigation pressure was maintained at approximately 40 cmH₂O. In suction-assisted procedures, controlled irrigation inflow was combined with regulated negative-pressure outflow to maintain adequate visibility while minimizing intrarenal pressure elevation.

Stone lithotripsy was performed using the “painting technique” [12], with laser pulses delivered in long-pulse mode to optimize dusting efficiency and laser–stone interaction. At the conclusion of the procedure, residual fragments were retrieved when necessary, using nitinol baskets, and a ureteral stent was placed according to predefined clinical indications and surgeon judgment.

### Data collection and outcomes

Demographic data including age, sex, body mass index, ASA score, were collected for all patients. Stone characteristics included anatomical location, number, maximum diameter, total stone volume, radiological density (Hounsfield units), and postoperative stone composition when available. Stone location was categorized as renal only, ureter only, or complex stone burden. Complex stones were defined as stones involving both the kidney and ureter or presenting as multiple/complex renal stone burden (e.g., partial or complete staghorn stones).

Preoperative stone volume was quantified using manual three-dimensional volumetric segmentation of non-contrast CT (NCCT) images using the dedicated 3D Slicer software [13]. All segmentations were conducted by a single trained investigator (C.G.G.) to ensure methodological consistency across centers. Segmentation was conducted using a standardized threshold-based approach with manual contour refinement when necessary. CT-based volumetric segmentation has been previously validated for accurate stone volume assessment in both experimental and clinical settings [14, 15]. Data was extracted at each center using a standardized data collection template. In patients presenting with complex stones, defined as stone burdens involving more than one intrarenal space, total stone burden was calculated as the sum of all segmented stone volumes per patient.

Intraoperative parameters, including pulse energy (J), pulse frequency (Hz), total power (W), total delivered energy (kJ), and laser-on time (LOT), were recorded, along with operative time and postoperative complications. Complications were classified according to the Clavien–Dindo classification system [16]. Laser settings were selected at the discretion of the operating surgeon, reflecting routine clinical practice; therefore, modulation-specific analyses were not performed.

### Outcomes

The primary outcome of the study was volumetric laser efficiency, assessed using standardized efficiency metrics as previously defined [17]. These metrics were calculated using pre- and postoperative segmented stone volumes. Ablation speed (mm³/s) was defined as ablated stone volume divided by laser-on time, and energy consumption (J/mm³) was defined as total delivered energy divided by ablated stone volume. These outcomes were calculated among patients who had full data on preoperative and postoperative segmentation data.

Secondary outcomes included SFR, perioperative complications, and the need for additional stone-related interventions.

Postoperative SFR was assessed 1–3 months after surgery according to institutional follow-up protocols. When available, NCCT with 1–2 mm slice thickness was used for radiological assessment. In patients without postoperative NCCT (*n* = 59), SFR was determined based on intraoperative endoscopic assessment. To account for potential verification bias, SFR outcomes were analyzed separately according to the assessment modality. For clarity, SFR was described in accordance with prior literature [18]. In patients with postoperative NCCT imaging: Grade A (Zero fragment rate), no visible residual fragments; Grade B (relative stone-free), residual fragments ≤ 2 mm; and Grade C (relative stone-free), residual fragments ≤ 4 mm. Patients with residual fragments > 4 mm were classified as not stone-free.

Additional stone-related treatment was defined as any secondary procedure performed for residual stones during follow-up, including both planned staged procedures and unplanned retreatment. The dataset did not allow reliable differentiation between planned and unplanned secondary procedures, and this distinction could therefore not be analyzed.

### Statistical analysis

All analyses were conducted in accordance with established statistical recommendations [19]. Continuous variables were summarized as medians and interquartile ranges (IQRs), and categorical variables as frequencies and percentages.

Group comparisons were performed using the Wilcoxon rank-sum test for continuous variables, and Fisher’s exact or Pearson’s Chi-square tests for categorical variables, as appropriate.

Violin plots were used to plot the changes in preoperative and postoperative stone volume. Additionally, box plots were used to represent the ablation speed calculated as the volume ablated for each second (mm^3^/s) according to stone type and composition. Given the lack of this data for the majority of patients, these results were only considered as exploratory. Stone-free rates and Zero fragment rate (ZFR) in accordance with the guidelines (Grade A, B and C) were indicated using proportions.

Predefined subgroup analyses were conducted according to anatomical stone location and imaging modality, and descriptive comparisons were used to assess potential variability across clinical scenarios. Given the moderate sample size and the large number of technical covariates, multivariable adjustment was considered potentially unstable and exploratory; therefore, findings are interpreted descriptively rather than causally.

Statistical significance was considered at *p* < 0.05. For all statistical analyses, R software environment for statistical computing and graphics was used (version 4.5.1; http://www.r-project.org/).

## Results

### Baseline characteristics

A total of 167 patients met the inclusion criteria, of whom 70 (42%) were female. The median age was 55 years (IQR 40–68), and the median BMI was 26.0 kg/m² (IQR 22.9–30.8). A history of urolithiasis was present in 17 patients (12%), and anatomical abnormalities were noted in 30 patients (18%).

Stone distribution was heterogeneous: 42 patients (25%) had isolated renal stones, 46 (28%) had ureteral stones only, and 79 (47%) presented with multiple anatomical locations. The median number of stones was 3 (IQR 1–3). Median stone density was 1579 HU (IQR 1148–1818), and the median preoperative stone volume was 987 mm³ (IQR 329–3124). Full baseline characteristics are summarized in Table 1.

**Table 1:** Baseline characteristics of 167 patients treated with p-Tm: YAG lithotripsy across three tertiary centers (2023–2025)

Characteristics	No. of patients (%)
No. of patients	167 (100)
Age, years	
Median (IQR)	55 (40–68)
Sex	
Female	70(42)
BMI, Kg/m^2^	
Median (IQR)	26 (23–31)
ASA score	
0	89(72)
1	31(25)
2	3(2.4)
Unknown	44
History of prior urolithiasis	17(12)
Anatomic anomaly	30(18)
Side	
Right	65(45)
Left	77(53)
Bilateral	4(2.7)
Unknown	21
*Stone location*	
Kidney only	42(25)
Ureter only	46(28)
Multiple locations	79(47)
Number of stones	
Median (IQR)	3 (1–3)
Maximum stone density, Hounsfield	
Median (IQR)	1579 (1148–1818)
Preoperative stone max diameter, mm	
Median (IQR)	13 (9–20)
Preoperative stone volume, mm^3^	
Median (IQR)	987 (329–3124)

*IQR *interquartile range, *ASA* American society of anesthesiologists

### Laser and performance efficiency outcomes

The overall median laser-on time (LOT) was 21.4 min (IQR 11–40). Median pulse energy, frequency, and power were 0.8 J (IQR 0.6–0.8), 15 Hz (IQR 10–20), and 10 W (IQR 6–15), respectively (Table 2). Laser settings were comparable across centers.

**Table 2:** Perioperative data and surgical outcomes

Characteristics	No. patients (%) [*N* = 167 (100)]
	Values (Median [IQR])
Laser on time, min	21.39 (11–40)
Laser settings	
Energy (J)	0.8 (0.6–0.8)
Pulse rate (Hz)	15 (10–20)
Power (W)	10 (6–15)
Pulse mode*	Fragmentation: 126 (75%); Popcorning: 3 (1.8%); Captive fragmentation: 11 (6.6%); Combined approach: 1 (1.8%); Unknown: 26 (16%)
Total delivered energy (kJ)	12 (5–30)
Energy consumption, J/mm^3^	13 (8–23)
Unknown	59
Ablation speed (mm ^3^/s)	0.55 (0.30–0.95)
Unknown	59
Postoperative stenting	163 (98)
Postoperative volume, mm^3^	
Median (IQR)	0 (0–777)
Unknown	59
UAS	
No	85 (51)
Traditional UAS	48 (29)
FANS	22 (13)
Missing	12 (7)
Complication grading	
Clavien 1–2	8 (5)
Clavien 3–4	1 (1)
Further stone surgery	45(27)

*J* Joule,* Hz *Hertz, *W* Watt, *UAS* ureteral access sheath, *FANS* Flexible aspirating navigable access sheath

* The variable “pulse mode” refers to the lithotripsy strategy selected and recorded intraoperatively on the laser platform (fragmentation, popcorning, captive fragmentation, or combined approach). In cases labeled as “Unknown,” the surgical strategy was not explicitly recorded in the operative report; however, detailed laser parameters (energy, frequency, power, total energy, and laser-on time) were available for all patients and were used for efficiency analyses

When lithotripsy was performed exclusively in the ureter, the median LOT was 15 min (IQR 6–23), with median pulse energy 0.8 J (IQR 0.60–0.80), frequency 10 Hz (IQR 5–20), and power 8 W (IQR 5–16). For kidney-only cases, the median LOT was 19 min (IQR 9–27), with median pulse energy 0.8 J (IQR 0.6–1), frequency 10 Hz (IQR 10–20), and power 10 W (IQR 6–14). In complex or multiple-location cases, the median LOT was 36 min (IQR 19–60), with pulse energy 0.8 J (IQR 0.6–1.0), frequency 15 Hz (IQR 10–20), and power 12 W (IQR 8–15) (Table 3).

**Table 3:** Laser settings and lasering efficiency stratified by stone location (kidney, ureter, multiple/complex)

Characteristics	Stone location [values (median [IQR])]		
	Kidney (*n* = 42)	Ureter (*n* = 46)	Multiple locations (*n* = 79)
Laser on time, min	19 (9–27)	15 (6–23)	36 (19–60)
Laser settings			
Energy (J)	0.8 (0.6–1.0)	0.8 (0.6–0.8)	0.8 (0.60–1.0)
Pulse rate (Hz)	10 (10–20)	10 (5–20)	15 (10–20)
Power (W)	10 (6–14)	8 (5–16)	12 (8–15)
Pulse mode	Dusting (long pulse): 31 (74%) Popcorning: 1 (2%) Unknown: 10 (24%)	Dusting (long pulse): 42 (91%) Captive Fragmentation: 2 (4.3%) Unknown: 2 (4.3%)	Dusting (long pulse): 53 (67%) Popcorning: 2 (2.5%) Captive Fragmentation: 9 (11%) Combined approach: 1 (1.3%) Unknown: 14 (18%)
Total delivered energy (KJ)	10 (3–21)	6 (2.7–20)	20 (11–40)
Unknown	12	20	25
J/mm ^3^	17 (8–26)	16 (10–20)	12 (7–18)
Unknown	12	20	25
Ablation speed (mm ^3^/s)	0.52 (0.30–0.79)	0.30 (0.21–0.58)	0.66 (0.43–1.09)
Unknown	12	20	25

The distribution of laser settings according to anatomical location is summarized in (Table 3). Dusting or long-pulse modes predominated in the ureter (91%), renal (74%), and multiple–location cases (67%).

The overall median total energy delivered was 12 kJ (IQR 5–30).

Among patients with known postoperative NCCT (*n* = 108), laser efficiency analysis showed a median energy consumption of 13 J/mm³ (IQR 8–23) and a median ablation speed of 0.55 mm³/s (IQR 0.30–0.95).

**Fig. 2:**
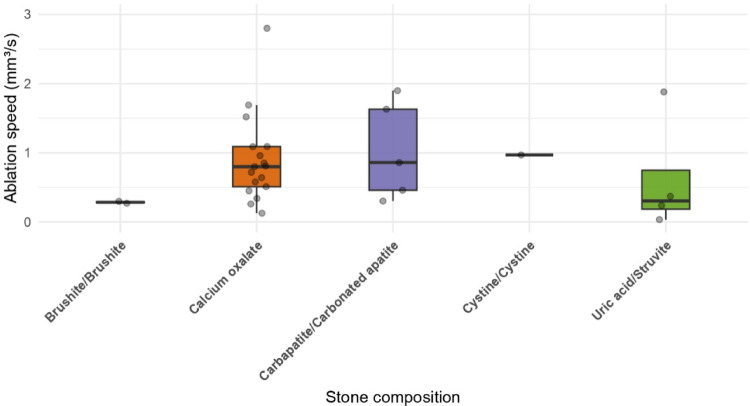
Ablation speed according to stone composition. Violin and box plots illustrating ablation speed (mm³/s) across stone compositions (calcium oxalate, brushite, carbapatite, cystine, uric acid/struvite)

Stone composition data were available for 53 patients with known postoperative NCCT, allowing exploratory composition-stratified analysis (Fig. 2). Median ablation speeds were 0.75 mm³/s (IQR 0.45–1.09) for calcium oxalate stones (*n* = 42), 0.29 mm³/s (IQR 0.28–0.30) for brushite stones (*n* = 6), and 1.25 mm³/s (IQR 0.58–1.93) for carbapatite stones (*n* = 12).

### Postoperative stone-free rates and complications

**Fig. 3:**
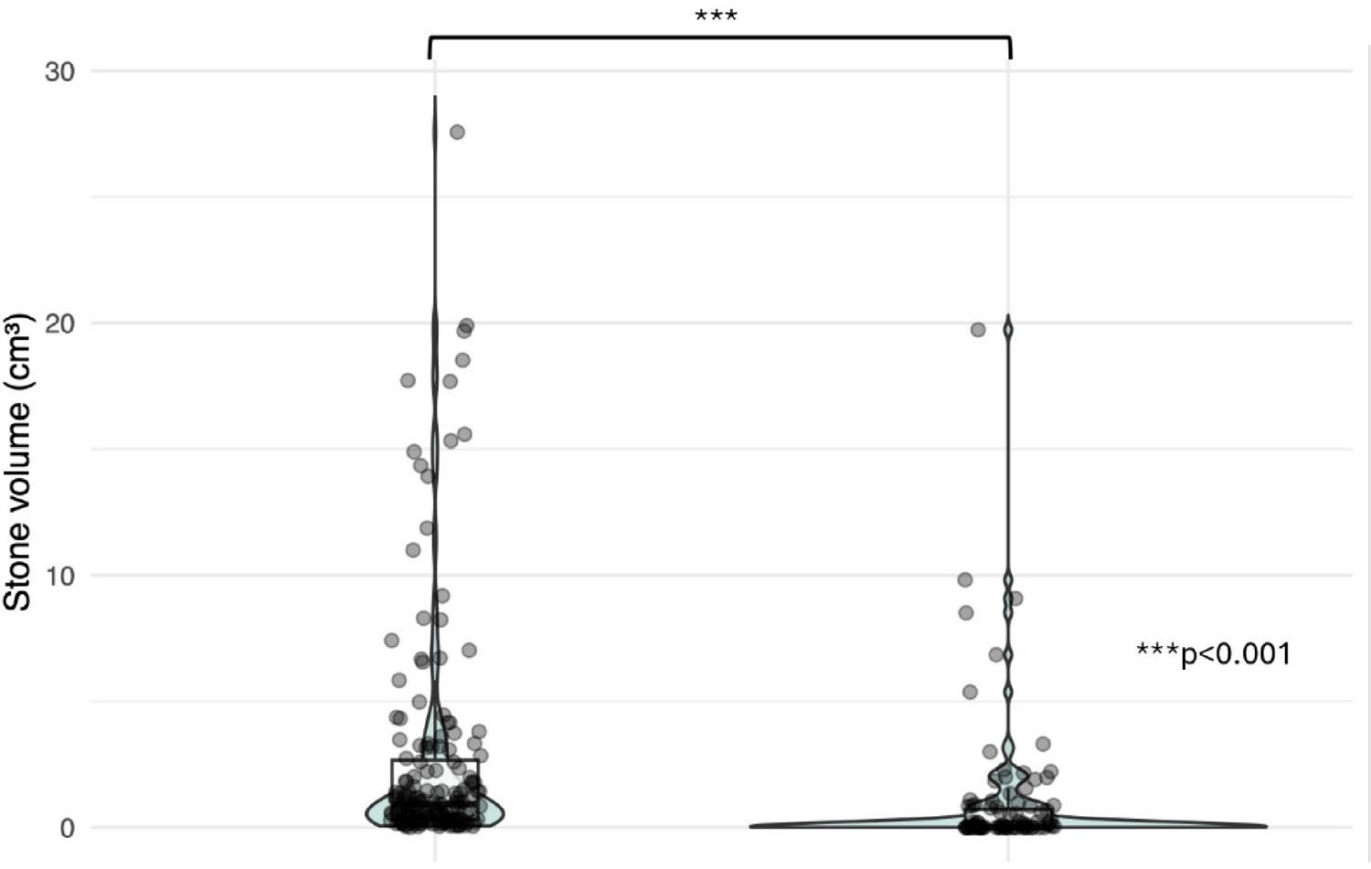
Preoperative versus postoperative segmented stone volumes Violin plots comparing stone volume (cm³) before and after flexible ureteroscopy with p-Tm: YAG laser. Median postoperative volume was 0 cm³; reduction was statistically significant (*p* < 0.001, Wilcoxon paired test)

Median postoperative stone volume was 0 mm³ (IQR: 0–777), corresponding to a median volume reduction of 781 mm³ (IQR: 236–2176) (Fig. 3).

#### Radiological stone-free rate outcomes (NCCT subgroup)

Postoperative radiological SFR outcomes were assessed in the subgroup of patients who underwent postoperative NCCT (*n* = 108), which represented the principal efficacy population for SFR analysis. Grade A, Grade B, and Grade C SFR outcomes were 47% (51/108), 59% (64/108), and 66% (71/108), respectively.

Stone-free outcomes varied according to stone location and complexity. In patients with isolated ureteral stones, Grade A SFR was achieved in 100% (26/26) of cases. In patients with isolated renal stones (*n* = 30), Grade A, B, and C SFR were 59%, 72%, and 79%, respectively. In contrast, multiple-location (*n* = 54) cases demonstrated lower success rates, with Grade A, B, and C SFR of 29%, 38%, and 46%, respectively. Stone burden was strongly associated with treatment success: patients with a preoperative stone volume ≤ 1,000 mm³ (*n* = 87) achieved Grade A, B, and C SFR of 75%, 87%, and 92%, respectively, whereas among patients with a stone burden ≥ 10,000 mm³ (*n* = 17), only 10% (*n* = 2) achieved Grade C SFR.

#### Endoscopic assessment (no postoperative NCCT)

Patients without postoperative NCCT follow-up had baseline characteristics comparable to those of patients who underwent postoperative CT imaging (sTable 1). In 59 patients (35%), postoperative NCCT was not available, and SFR was therefore assessed by intraoperative endoscopic evaluation only. In this subgroup, complete stone clearance was visually assessed in 20 patients (34%), while residual fragments were observed in 39 patients (66%).

#### Additional treatments and complications

Overall, 45 patients (27%) required additional stone-related treatment during follow-up. In particular, 35% of patients in the NCCT missing subgroup underwent further stone treatment compared to 22% of the NCCT available subgroup (*p* = 0.063).

Postoperative morbidity was low: Clavien–Dindo grade I–II complications occurred in eight patients (4.8%), and one patient (0.6%) experienced a grade III complication. No grade IV–V adverse events were observed.

## Discussion

The present multicenter cohort study evaluated the real-world clinical performance and volumetric laser efficiency of the p-Tm: YAG laser during FURS across three tertiary referral endourology centers. Using standardized pre- and postoperative volumetric segmentation through dedicated software, we assessed ablation speed (mm^3^/s) and energy consumption (J/mm³) in a cohort representative of contemporary endourological practice. This study was designed as a non-comparative performance evaluation to provide complementary clinical evidence regarding the efficiency and safety profile of this emerging laser technology. By applying CT-based volumetric segmentation in a multicenter clinical setting, the present study extends this methodology beyond bench testing into routine practice. This approach enables efficiency metrics to be interpreted alongside SFR outcomes and safety, representing a key methodological strength.

In this cohort of 167 patients, p-Tm: YAG demonstrated measurable lithotripsy efficiency. Median pulse energy was 0.8 J across anatomical locations, with median pulse frequency of 10 Hz for ureteral and renal stones and 15 Hz for complex located stones. Corresponding median power ranged from 8 W for ureteral stones to 12 W for complex cases. The overall median ablation speed was 0.55 mm^3^/s, with lower efficiency observed in ureteral stones (0.3 mm^3^/s) and higher values in renal (0.52 mm^3^/s) and multiple–location stones (0.66 mm^3^/s). These values reflect the efficiency of stone ablation in routine clinical practice despite procedural variability, heterogeneous stone composition, and complex anatomical conditions.

Exploratory analyses suggested variability in ablation speed according to stone composition. Calcium oxalate stones demonstrated expected ablation behavior, whereas brushite exhibited lower efficiency consistent with its known resistance to endoscopic laser lithotripsy [20]. Differences observed for other compositions should be interpreted cautiously given limited sample sizes and the absence of multivariable adjustment.

The ablation efficiency observed in this clinical cohort is consistent with findings reported in experimental and translational studies. In vitro investigations by Quarà et al. reported p-Tm: YAG ablation rates ranging from 0.38 to 0.45 mm³/s depending on stone composition and laser settings. Specifically, ablation rates of 0.38 mm³/s at 0.5 J–20 Hz and 0.42 mm³/s at 1 J–10 Hz were observed for hard stones, while softer stones demonstrated slightly higher ablation rates under similar conditions [21].

Our study reported a median energy consumption of 13 J/mm^3^. In vitro, Moretto et at. demonstrated that energy consumption per unit volume is strongly influenced by both pulse energy and stone composition. At 0.5 J laser setting, the energy required to ablate calcium oxalate monohydrate stones was 30 J/mm³, compared with 76 J/mm³ for cystine, and 23 J/mm³ for uric acid stones. Increasing pulse energy improved ablation efficiency for each stone composition, reducing energy consumption to 10–16 J/mm³ at 0.8 J, and to 7 J/mm³, 8 J/mm³, and to 4–8 J/mm³ respectively at 1 J [22]. The median energy consumption of 13 J/mm³ observed in our cohort falls within this experimentally predicted range, despite the increased complexity of in vivo lithotripsy, which includes fragment displacement, endoscopic maneuvering, and heterogeneous stone composition.

Clinical series evaluating p-Tm: YAG reported comparable efficiency metrics. Panthier et al. reported ablation efficiency of 14.8 J/mm^3^ and ablation speed of 0.75 mm³/s, with SFR of 95% in a cohort primarily composed of renal stones. Consistently, we reported variations of energy efficiency according to anatomical location. In our cohort, the lower ablation rates were recorded for kidney and ureteral stones − 17 and 16 J/mm^3^, respectively- while multiple located lithiasis reported the most efficient energy dissipation with an ablation rate of 12 J/mm^3^. The discrepancy between isolated kidney stones and complex stone burden likely represents the variations in fragment mobility, and irrigation dynamics and stone composition. Indeed, as reflected by the similar ablation rate between these categories, complex stones frequently consist of infection-related compositions, such as struvite, which demonstrate higher susceptibility to laser energy and may account for the observed differences in lithotripsy efficiency [23].

Considering the in vivo series using the same p-Tm: YAG, Kutchukian et al. reported a median ablation speed of 0.69 mm³/s and median energy consumption 14 J/mm³, with a SFR and ZFR of 79 and 50%, respectively, for a median stone volume of 1239 mm³ [24]. Similarly, in our cohort, we observed a ZFR of 47% and a SFR of 66% for renal stones with comparable stone burden. These findings support the ability of the p-Tm: YAG to achieve effective stone clearance during FURS, with complete clearance obtained in nearly half of patients undergoing FURS. This rate may be underestimated, as some patients without postoperative NCCT demonstrate complete endoscopic clearance with visual confirmation of residual dust only [25].

Complex lithiasis demonstrated lower success rates, with Grade A, B and C SFR of 29%, 38%, and 46%, respectively. These findings suggest that stones involving multiple intrarenal locations or involving both the kidney and the ureter might present increased procedural complexity and are more likely to require staged or second-look procedures to achieve complete clearance.

The clinical importance of obtaining the ZFR has been already highlighted by Werth et al., who demonstrated that patients with residual fragments between 1 and 4 mm experienced significantly higher rates of stone-related events at six months, including complication and retreatment [26]. Our findings are consistent with this observation, with overall complications rates of 5% and high-grade complications in only 1% of patients. Although the retreatment rate in our cohort was 27%, this result has not been considered among our main outcomes. Indeed, in many cases, secondary procedures were planned as part of staged management strategies in patients with large or complex stone burdens rather than reflecting procedural failure.

The outcomes of p-Tm: YAG have been provided also against the other laser systems. In vitro comparison between p-Tm: YAG and Ho: YAG under standardized laboratory conditions have reported a significantly more efficient lithotripsy capabilities with improved ablation rate of p-Tm: YAG, in particular when increased pulse durations are applied [8, 27]. Similarly, comparable dusting performance between p-Tm: YAG and TFL systems under standardized conditions have been investigated. In vitro, no statistically significant difference in ablated stone mass was observed between p-Tm: YAG and TFL when identical pulse energy and frequency settings were applied [28]. In vivo, a clinical trial comparing TFL and p-Tm: YAG, patients treated with TFL lithotripsy presented a similar SFR, while ZFR was higher in the TFL group [29]. These results suggest how thulium-based technologies might be considered complementary rather than alternative in FURS. Evidence on the laser shape dynamic and energy interaction with different stone composition might clarify which system may be preferred in clinical practice for the treatment of different urological lithiasis conditions [30]. However, comparative clinical studies remain limited, and consistent prospective multicentric evidence of superiority among the expanding platforms available is lacking [31]. Accordingly, these findings should not be interpreted as evidence of superiority or equivalence to other laser platforms, but rather as real-world efficiency and safety data contributing to the evolving literature on thulium-based lithotripsy systems.

Some limitations should be acknowledged. First, the retrospective design introduces potential documentation variability and residual confounding. Second, procedural heterogeneity across centers reflects real-world practice but limits causal inference. Third, stone composition was available only in a subset of patients, and composition-specific analyses should therefore be considered exploratory. In addition, postoperative imaging modality and timing were not fully standardized. In 59 patients, postoperative NCCT was not available; therefore, radiological stone-free outcomes were primarily interpreted within the subgroup that underwent postoperative NCCT, which represented the principal efficacy population for stone-free assessment. Although baseline characteristics were comparable between patients with and without postoperative NCCT, suggesting that differences in follow-up were mainly related to institutional practice rather than patient characteristics, some degree of verification bias cannot be completely excluded. The rate of stone-related reintervention was 27%, which may appear relatively high; however, this figure included both unplanned retreatment and planned staged procedures in patients with large or complex stone burden. Because the database did not systematically distinguish between planned staged procedures and unplanned retreatment, this outcome should be interpreted with caution. Due to the small sample size, further substratification other than location in assessing laser efficiency and clinical outcomes for the use of UAS or FANS was not possible. Finally, the absence of a comparator arm precludes any conclusion regarding the relative performance of the p-Tm: YAG laser compared with established laser platforms.

## Conclusion

This multicenter real-world study provides volumetric performance data on the use of p-Tm: YAG during FURS across different clinical scenarios. In this cohort, the platform showed measurable ablation efficiency and was associated with acceptable safety and clinically useful stone clearance outcomes. These findings support the clinical feasibility of this technology in routine practice; however, due to the retrospective design and absence of a comparator arm, these results should be interpreted as descriptive real-world data rather than evidence of superiority or equivalence. Prospective comparative studies are warranted to further define its relative effectiveness and optimal clinical role.

## Supplementary Information

Below is the link to the electronic supplementary material.

Supplementary Material 1

## Data Availability

The data that support the findings of this study are available from the corresponding author, Carlos Gonzalez Gonzalez, upon reasonable request.

## Abbreviations

ASA: American society of anesthesiologists
BMI: Body mass index
CM^3^: Cubic centimeters
CT: Computed tomography
FANS: Flexible active negative suction
Fr: French gauge
FURS/fURS: Flexible ureterenoscopy
HU: Hounsfield units
IQR: Interquartile range
J: Joules
J/mm^3^: Energy consumption per ablated stone volume
kJ: Kilojoules
LOT: Laser-on time
mm: Millimeter
mm^3^: Cubic millimeter
mm^3^/s: Ablation speed
NCCT: Non-contrast computed tomography
PCNL: Percutaneous nephrolithotomy
PEARLS: Progressive endourological association for research and leading solutions
p-Tm:YAG: Pulsed thulium:yttrium–aluminium–garnet laser
RIRS: Retrograde intrarenal surgery
SER: Stone-free rate
TEL: Thulium fiber laser
UAS: Ureteral access sheath
W: Watt
ZER: Zero-fragment rate
