# Supplementary material for: Flexible ureterorenoscopy and lithotripsy with pulsed thulium: YAG laser: a multicenter retrospective study
Source: World J Urol. 2026 May 24;44(1):383. doi: 10.1007/s00345-026-06467-1 (PMC13199192; doi:10.1007/s00345-026-06467-1)
Supplement: Supplementary file 1 — Supplementary Material 1 [file 345_2026_6467_MOESM1_ESM.docx]

**sTable 1.** Baseline characteristics of 167 patients treated with p-Tm:YAG lithotripsy across three tertiary centers (2023–2025) stratified for NCCT follow-up status.

| **Characteristics** | **No. of patients (%)** | | **p value** |
| --- | --- | --- | --- |
| **No. of patients** | NCCT | No NCCT |  |
|  | 108 (100) | 59 (100) |  |
| **Age, years** |  |  | 0.2 |
| Median (IQR) | 56 (45 - 68) | 53 (36 – 66) |  |
| **Sex** |  |  | 0.7 |
| Female | 46(43) | 24(41) |  |
| **BMI, Kg/m^2^** |  |  | 0.4 |
| Median (IQR) | 26 (23 - 30) | 26 (23 - 32) |  |
| **ASA score** |  |  | 0.7 |
| 0 | 60(71) | 29(76) |  |
| 1 | 22(26) | 9(24) |  |
| 2 | 3(3.5) | 0(0) |  |
| Unknown | 23 | 21 |  |
| **History of prior urolithiasis** | 11(10) | 6(11) | 0.6 |
| **Anatomic anomaly** | 20(19) | 10(17) | 0.5 |
| **Side** |  |  | 0.11 |
| Right | 51(47) | 14(37) |  |
| Left | 53(49) | 24(63) |  |
| Bilateral | 4(3.7) | 0 (0) |  |
| Unknown | 0 | 21 |  |
| **Stone location** |  |  | 0.093 |
| Kidney only | 30(28) | 12(20) |  |
| Ureter only | 26(24) | 20(34) |  |
| Multiple locations | 52(48) | 27(46) |  |
| **Number of stones** |  |  | 0.15 |
| Median (IQR) | 2 (1-3) | 3 (1-3) |  |
| **Maximum stone density, Hounsfield** |  |  | 0.6 |
| Median (IQR) | 1,582 (1,277 - 1,803) | 1,545 (959 - 1,818) |  |
| **Preoperative stone max diameter, mm** |  |  | 0.6 |
| Median (IQR) | 13 (10 - 20) | 13 (9 - 21) |  |
| **Preoperative stone volume, mm^3^** |  |  |  |
| Median (IQR) | 927 (280 - 2,011) | 1,170 (407 - 3,798) | 0.095 |
